# A Structural Approach into Drug Discovery Based on Autophagy

**DOI:** 10.3390/life11060526

**Published:** 2021-06-04

**Authors:** Sung-Min Kang, Do-Hee Kim

**Affiliations:** 1 College of Pharmacy, Duksung Women’s University, Seoul 01369, Korea; smkang@duksung.ac.kr; 2 College of Pharmacy, Jeju National University, Jeju 63243, Korea; 3 Interdisciplinary Graduate Program in Advanced Convergence Technology & Science, Jeju National University, Jeju 63243, Korea

**Keywords:** autophagy, protein structure, drug discovery

## Abstract

Autophagy is a lysosome-dependent intracellular degradation machinery that plays an essential role in the regulation of cellular homeostasis. As many studies have revealed that autophagy is related to cancer, neurodegenerative diseases, metabolic diseases, and so on, and it is considered as a promising drug target. Recent advances in structural determination and computational technologies provide important structural information on essential autophagy-related proteins. Combined with high-throughput screening methods, structure-activity relationship studies have led to the discovery of molecules that modulate autophagy. In this review, we summarize the recent structural studies on autophagy-related proteins and the discovery of modulators, indicating that targeting autophagy can be utilized as an effective strategy for novel drug development.

## 1. Introduction

Autophagy is a lysosome-dependent intracellular degradation process machinery that plays an essential role in the regulation of cellular homeostasis [1,2]. Overabundant or malfunctioning cellular constituents such as misfolded proteins, damaged organelles, and invasive microorganisms undergo this degradative pathway [3]. Aberrant regulation of autophagy disturbs protein degradation and organelle turnover in mammalian cells, which eventually leads to abnormal cell growth, cell death, and protein accumulation, resulting in diverse human diseases [1]. Representative diseases affected by impaired autophagy include neurodegenerative diseases, cancer, metabolic diseases, infectious diseases, and others [4]. For the treatment of these diseases, many autophagy modulators, either inducers or blockers for each specific autophagy systems, have been discovered and studied [5]. Autophagy activators such as rapamycin derivatives, everolimus (mTOR inhibitor) [6,7], and nilotinib (AMPK activator) [8], and autophagy inhibitors such as bortezomib targeting the 38-MAPK-JUNK pathway [9], and idelalisib targeting phosphoinositide 3-kinase (PI3-K) [10] are US Food and Drug Administration (FDA)-approved modulators for cancer treatment; several other autophagy modulators are undergoing clinical trials.

Recent advances in structure determination and computational techniques have led to an understanding of the processes and mechanisms of autophagy. The structural and functional information on autophagy-related proteins could be utilized in the development of therapeutic modulators to treat the diseases related to autophagy. In this review, we summarize the currently determined structures of autophagy-related proteins and the state of modulator discovery based on their structures. These studies and the resulting information could contribute to the development of novel drugs with selectivity and efficacy in the disease.

## 2. General Information on Autophagy

**Figure 1:**
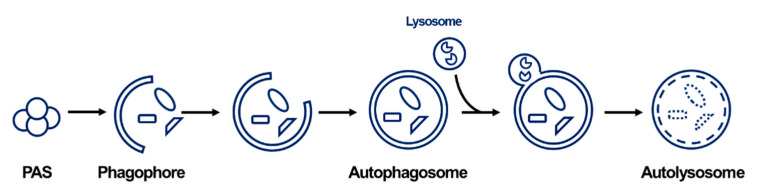
The overall process of autophagy in mammals (macrophagy). After the induction of autophagy by an external signal, PAS is formed. The recruited ATG proteins and lipids form phagophores with isolated membranes. Organelles and proteins engulfed by the membrane form a double-membrane vesicle, autophagosome. After the fusion with the lysosome, lysosomal hydrolases is released into the cargo of autophagosome and degrade its contents.

Autophagy includes the delivery pathway of cytosolic material to the lysosome in mammalian cells or the vacuoles in plant and yeast cells. Autophagy is largely classified into three types: macroautophagy, microautophagy, and chaperone-mediated autophagy (“autophagy” usually refers to macroautophagy) [11]. Since the discovery of autophagy in the buddying yeast *Saccharomyces cerevisiae*, the identification of autophagy-related genes (ATG) in yeast has promoted the characterization of the molecular mechanism of autophagy membrane dynamics [12,13]. Under normal conditions, autophagy is involved in the maintenance of cellular homeostasis. However, excess autophagy processes are induced by extracellular stimuli and stress. After autophagy induction, a series of processes continue as follows: initiation, nucleation-elongation-maturation, fusion, and degradation (Figure 1) [14,15]. In the initiation step, the pre-autophagosomal structure (PAS) sequesters the UNC-51-like autophagy-activating kinase 1 (ULK1) complex and ATG proteins after stimulation. Next, the recruited ATG proteins and lipids form phagophores. The bulk cytosol and/or organelles engulfed by the membrane form a double-membrane vesicle, namely the autophagosome that docks and fuses with the lysosome. At the final degradation step, the cargo inside the autolysosome is degraded and the resulting metabolites are recycled and reused in the cytoplasm. 

## 3. Structural Studies of ATG Proteins and Drug Discovery

### 3.1. ULK1

ULK1, a homolog of ATG1 in mammals, is involved in the initiation of the autophagy pathway by forming a complex with multiple regulatory subunits, such as FIP200, ATG13, and ATG101 [16]. As a serine/threonine (S/T) kinase in the core autophagy pathway, ULK1 phosphorylates many substrate proteins in the pro-autophagy signaling step. After the activation of the ULK1 complex, nucleation of the immature autophagosomes is facilitated [17]. Therefore, the inhibition of ULK1 is an attractive strategy for blocking the initial stage of autophagy. 

**Figure 2:**
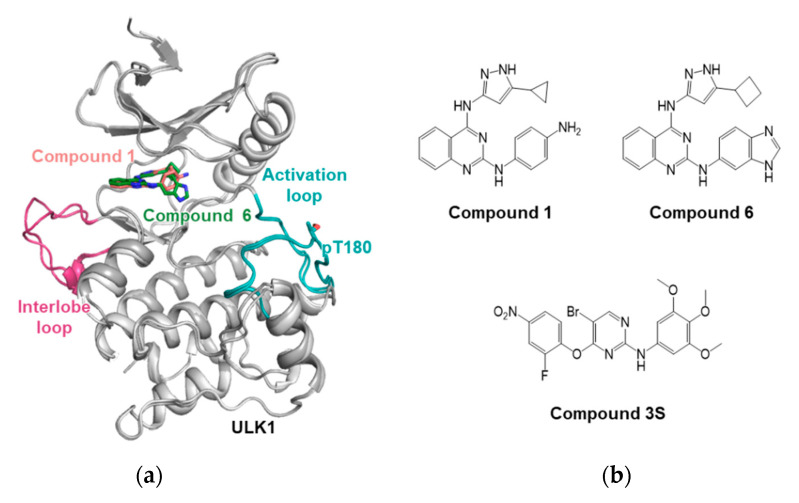
ULK1 and inhibitors. (**a**) Structural overlays of ULK1s, complexed with compounds 1 (salmon) and 6 (green). ULK1 structures are shown in gray color. The interlobe loop and activation loop with phosphorylated Thr180 are colored with magenta and turquoise, respectively. (**b**) Inhibitors for ULK1. Compounds 1 and 6 are shown in the upper panel. Two small molecules share a similar structural scaffold. Compound 3S is denoted at the lower panel. Protein and chemical structures were drawn by the programs PyMOL (The PyMOL Molecular Graphics System, Version 2.3.4 Schrӧdinger, LLC., Cambridge, MA, USA) and ChemDraw Pro20.1 (Perkin Elmer, Waltham, MA, USA), respectively.

The structure of ULK1 was first discovered by Lazarus et al. using inhibitors when developing a drug-targeting ULK1 [18]. As it was difficult to obtain crystals of apo ULK1, the complex structures of ULK1 were determined using two inhibitors: compound 1 (PDB ID: 4WNO) and compound 6 (PDB ID: 4WNP) (Figure 2a,b). The inhibitors against ULK1 were screened from a library of 764 compounds using a standard ^32^P-ATP radioactive assay with maltose-binding protein (MBP) as the substrate. Compound 1, possessing pyrazole aminoquinazolines, inhibited ULK1 with an IC_50_ of 160 nM, stabilizing the kinase domain of ULK1 and promoting its crystallization. The ULK1 structure possesses a conserved kinase fold and shares common features with the ULK2 structure, but it exhibits a unique property in the positively-charged long loop between the N- and C-terminal lobes. This loop is involved in substrate recognition and regulation of kinase activity [18]. On the basis of the crystal structure, a structure–activity relationship (SAR) study identified compound 6 with increased potency (IC_50_ of 8 nM). Although the compounds were potent, they were not highly selective against ULK1; various SAR studies have been conducted to improve their efficacy and selectivity. Lazarus et al. subsequently discovered an inhibitor with improved selectivity for ULK1 [19], an aminopyrimidine scaffold containing BX-795 that exhibited high selectivity for several kinases including as 3-phosphoinositide-dependent kinase 1 (PDK1), and was used as a new lead compound [20]. Furthermore, to obtain compounds with increased selectivity for ULK1, a series of substitutions around the BX-795 scaffold were introduced. Owing to SAR studies, smaller compounds with better potency against ULK1 were derived (compounds 2 and 3). The crystal structure of ULK1 with compound 3 (PDB ID: 5CI7) revealed the critical residues that exert the selectivity of the compound [19].

The upregulation of ULK1 in some cancer tissues has been reported, and the knockdown or the selective inhibition of ULK1 resulted in cell apoptosis in non-small cell lung cancer (NSCLC) cells [21]. To discover potential drug candidates for the NSCLC therapy, the pharmacophore modeling and SAR based on the ULK1 structure (PDB ID: 4WNO) were conducted by Sun et al. [22], resulting in the compound 3S having good inhibitory activity against tumor cells, and the ULK1 kinase was discovered (Figure 2b). It efficiently blocked autophagy by inhibiting ULK1 and promoted apoptosis in human lung carcinoma A549 cells.

### 3.2. Autophagy-Specific Lipid Kinase Vacuolar Protein Sorting 34 (VPS34)

VPS34, a PI3K class III isoform, consists of class III PI3K complex I with VPS15, Beclin1, and ATG15L. This complex is involved in phagophore formation during the nucleation step [23,24]. VPS34 catalyzes the phosphorylation of phosphatidylinositol (PtdIns), resulting in phosphatidylinositol 3-phosphate (PtdIns3P) [23,24]. Therefore, the PtdIns3P-binding protein participates in the formation of autophagosomes [25]. On the basis of this key role in the autophagy machinery, VPS34, involved in vacuolar protein sorting, is also required for vesicle trafficking. Several studies have revealed the potential roles of VPS34 in cell proliferation and cancer [26,27,28,29]. This is an attractive target because VPS34 is involved in the nucleation step, and VPS34 inhibitors could block the early stage of autophagy. Several VPS34 inhibitors have been studied and discovered. 

**Figure 3:**
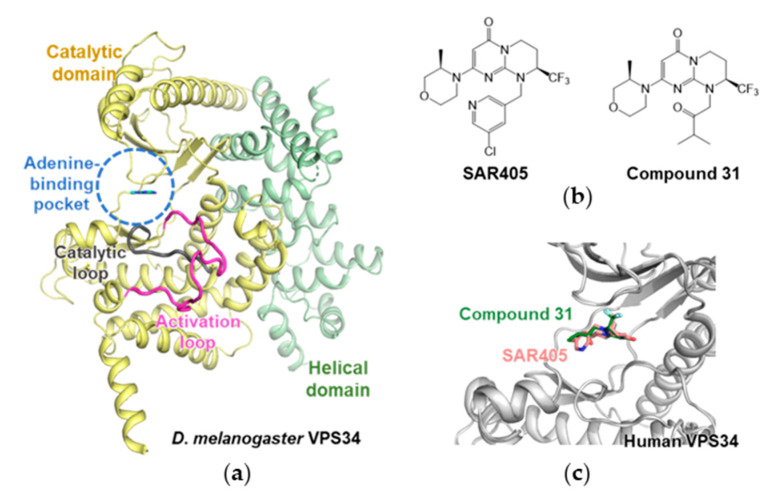
Structure of VPS34s. (**a**) VPS34 from *D*. *melanogaster* with 3-methyladenine (MA) (PDB ID: 2X6F). 3-MA represented in cyan color is positioned in an adenine-binding pocket. Catalytic and helical domains are colored in yellow and light green, respectively. Catalytic and activation loops are highlighted in dark gray and magenta, respectively. (**b**) Human VPS34 inhibitors SAR405 and compound 31. (**c**) Human VPS34 with inhibitor. The structure of human VPS34 is shown in gray color. Compound 31 and SAR405 are positioned similarly and are colored in green and salmon, respectively.

In these studies, the first crystal structure of VPS34 lacking the C2 domain from *Drosophila melanogaster* was determined to possess a constricted adenine-binding pocket (PDB ID: 2x6H) [30]. The overall structure of VPS34 comprises a helical domain and a catalytic domain. The distinct feature of the enzyme is the ordered phosphoinositide-binding (activation) loop (Figure 3a). In this study, several complex structures with autophagy inhibitors were investigated. On the basis of these structural insights, more potent and specific VPS34 inhibitors have been developed.

High-throughput screening (HTS) and structure-based design chemical optimization have resulted in selective inhibitors against VPS34, such as SAR405 and other compounds [31,32]. SAR405 is a selective ATP-competitive inhibitor with an IC_50_ of 1 nM in the phosphorylation of a PtdIns substrate by human recombinant VPS34 enzyme (Figure 3b) [32]. A series of biochemical assays supported the selectivity of SAR405 for VPS34. For further understanding, the crystal structure of human VPS34 with SAR405 (PDB ID: 4OYS) was determined (Figure 3c), providing information on the chemical optimization and selectivity improvement of VPS34 inhibitors. Furthermore, SAR405 inhibits autophagy induced by mTOR and synergizes with the US FDA-approved mTOR inhibitor everolimus. These synergetic effects result in the inhibition of cell proliferation in renal tumor cell lines.

The VPS34 inhibitor was also considered for the treatment of solid tumors and examined via HTS by Pasquier et al. [33]. The authors reported tetrahydropyrimidopyrimidinone derivatives. The complex structure of human VPS34 and the resulting compounds revealed an Asp-Phe-Gly (DFG) in the conformation of inhibitors. Compound 31, which exhibits nanomolar biochemical and cellular activities against VPS34, was derived through multiparametric chemical optimization (Figure 3b). Compound 13 exhibited selectivity with an IC_50_ of 2 nM for VPS34. The crystal structure of human VPS34 with compound 13 (PDB ID: 4UWL) also showed the role of the morpholine synthon in high selectivity against class I PI3Ks (Figure 3c). These studies have provided insights into the development of selective and potent drugs for cancer therapy based on the VPS34.

### 3.3. ATG8-ATG3

ATG8, a ubiquitin-like protein, is an essential component of the autophagy mechanism and binds to the membrane lipids [34,35]. ATG8 is also vital for the formation and elongation of the autophagosomes and is essential to the process of autophagy [36]. Autophagy has been attracting attention as a novel target in numerous critical diseases, from cancer to eukaryotic parasitic infections [37]. Although the exact function of ATG8 is not fully understood, it appears to be essential for autophagosome biogenesis and apicomplexan localization [38]. In this section, we discuss the techniques and compounds designed through computational screening of protein–protein interactions (PPIs). The resulting compounds mimic the ATG3 interaction motifs and share common scaffolds that are effective for disease-causing apicomplexans. 

**Figure 4:**
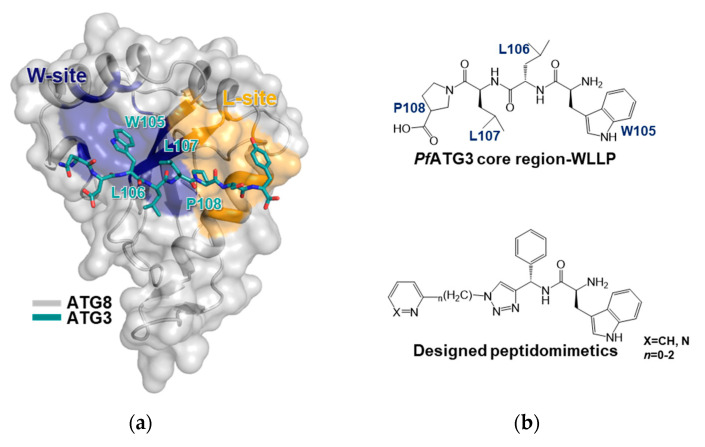
*P*. *falciparum* ATG8-ATG3 PPI inhibitor. (**a**) The overall structure of the ATG8–ATG3 complex. The core region of the ATG3 peptide is binding through the W-site (blue) and L-site (yellow) of ATG8. The key residues of the core region of ATG3 are labeled in W105-L106-L107-P108. (**b**) *Pf*ATG3 core region represented in peptide structure (upper) and designed peptidomimetics (below).

Hain et al. first discovered the protein structure of ATG8; it interacts with ATG3 (PDB ID: 4EOY) (Figure 4a) [39]. ATG3 and ATG8 interact through the L- and W-sites of ATG8, and these interfaces handle the entire ATG3 [40]. Mutational studies were conducted on R27, E44, K45, K46, K47, and L49 of ATG8, which are the receptor sites for ATG3. The MBP pull-down assay revealed that R27, K47, and L49 dramatically reduced the binding affinity between ATG8 and ATG3. Multisite mutations on E44, E45, and K46 also demonstrated binding destabilization despite damage to structural integration. Furthermore, a cascading computational screening and a final 352 small molecule library were treated with surface plasmon resonance; it was concluded that the IC_50_ of the compound was less than 150 µM. This indicates that at least more than one interaction site between ATG8 and ATG3 could be a novel druggable region.

Hain et al. later found a more powerful inhibitor again [41]. They optimized the first scaffold into a standard functional platform. Here, the Medicines for Malaria Venture malaria box containing 200 drug-like and 200 probe-like molecules was screened. It was found that a common scaffold, 4-pyridin-2-yl-1,3-thiazol-2-amine, was incorporated as the N-substituent in various anilines or benzamide-containing compounds (Figure 4b). Importantly, it was revealed that L115 of ATG8 participates directly in the L-site interaction and decreases the volume of the drug-binding pocket. Thus, caution is required regarding the effects of single amino acid mutations on binding.

Hain et al. republished data on new therapies against malaria using a more well-suited mechanism utilizing cross-reactivity against disease-causing apicomplexans [42] with the virtual library screening (VLS) method using protein structure. Employing an in silico method in which compounds are computationally docked to a known protein structure, they used the X-ray structure of *P*. *falciparum* ATG8 (PDB ID: 4EOY) [39] as a template for VLS, focusing on the finding that ALC25, a potent inhibitor of *Pf*ATG3-*Pf*ATG8, does not affect the interaction of the human homolog, hATG3-hLC3.

More recently, Villa et al. finally developed new antimicrobial PPI inhibitors by applying computational techniques to the crystal structure of the ATG3 and ATG8 complex (PDB ID: 4EOY) [39,43]. The inhibitor is a peptidomimetic that mimics the ATG3 interaction motif and delays the blood and liver stages of parasite growth. It represented the inhibition of the ATG8–ATG3 interaction of *P*. *falciparum*. In concordance with the calculated predictions, the Trp-Leu-Leu-Pro (WLLP) sequence of *Pf*ATG3 was identified as the most suitable structure to be mimicked by new *Pf*ATG3-*Pf*ATG8 PPI inhibitors. The developed peptidomimetics mimic WLLP, the core region of *Pf*ATG3. Taken together, these structural and interaction studies provide insights for future drug discovery based on PPIs.

## 4. Conclusions

Many studies have been conducted over the last several decades to elucidate the mechanism of autophagy. Structural studies on autophagy-related proteins have focused on the related mechanisms of human disease. Depending on the disease, the mechanisms involved in autophagy are different. Therefore, potential strategies should be based on the given diseases for the selective targeting. In this mini review, we summarized the representative examples of drug discovery based on the structures of the autophagy-related proteins. Following the example of ATG8-ATG3, the development of PPI inhibitors in autophagy will lead to the development of novel drugs that have not been previously studied. Binding sites mimicking peptides or cascade derivatives of peptidomimetics could also provide a useful example. Small molecules explored via HTS and virtual screening with SAR studies may also be effective strategies for drug discovery in ULK1 and VPS34. In addition to ULK1, VPS34, and ATG8-ATG3, there are other key human ATG proteins involved in the autophagy machinery, the structures of which have been elucidated, for example, ATG3, ATG4B, ATG5, and ATG12 [44,45,46]. The use of this important structural information is expected to enable the development of many novel modulators with improved efficient druggability.
